# Supplementary material for: Complex‐centric proteome profiling by SEC‐SWATH‐MS
Source: Mol Syst Biol. 2019 Jan 14;15(1):e8438. doi: 10.15252/msb.20188438 (PMC6346213; doi:10.15252/msb.20188438)
Supplement: Supplementary file 7 — Dataset EV6 [file MSB-15-e8438-s007.zip › feature_plots_bioplex/P78508.pdf]

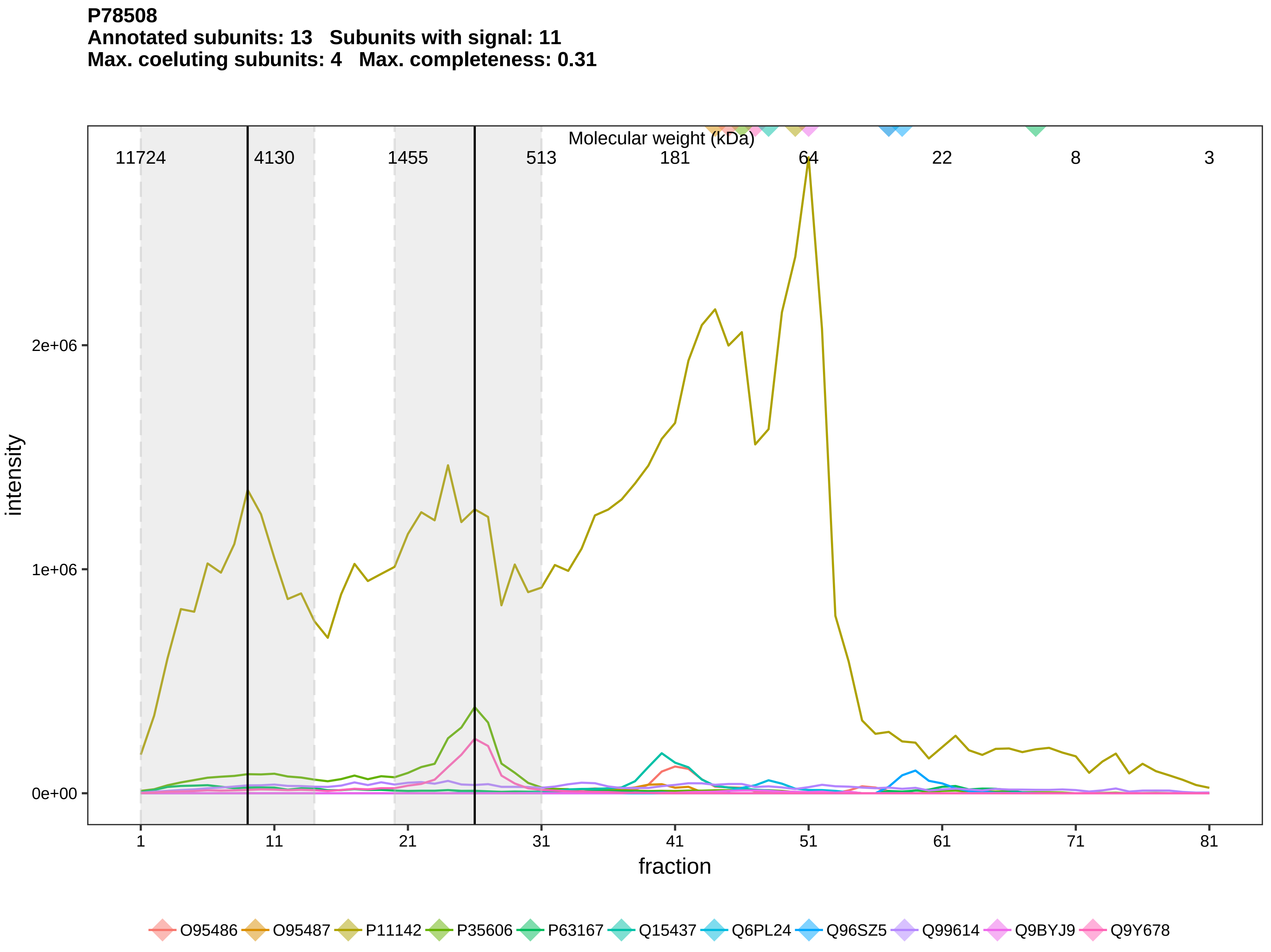
P78508
Annotated subunits: 13 Subunits with signal: 11
Max. coeluting subunits: 4 Max. completeness: 0.31
Molecular weight (kDa)
11724
4130
1455
513
181
64
22
8
3
intensity
2e+06
1e+06
0e+00
1
11
21
31
41
51
61
71
81
fraction
O95486
O95487
P11142
P35606
P63167
Q15437
Q6PL24
Q96SZ5
Q99614
Q9BYJ9
Q9Y678
